# Clinical profile and treatment outcomes of idiopathic intracranial hypertension: a multicenter study from Korea

**DOI:** 10.1186/s10194-024-01794-3

**Published:** 2024-06-25

**Authors:** Kyung-Hee Cho, Seol-Hee Baek, Sung-Hee Kim, Byung-Su Kim, Jong-Hee Sohn, Min Kyung Chu, Mi-Kyoung Kang, Hee Jung Mo, Sang-Hwa Lee, Hong-Kyun Park, Soohyun Cho, Sun-Young Oh, Jong-Geun Seo, Wonwoo Lee, Ju-Young Lee, Mi Ji Lee, Soo-Jin Cho

**Affiliations:** 1 Department of Neurology, Korea University Anam Hospital, Korea University College of Medicine, Seoul, Republic of Korea; 2 Department of Neurology, Ewha Womans University Mokdong Hospital, Ewha Womans University School of Medicine, Seoul, Republic of Korea; 3 Department of Neurology, Chuncheon Sacred Heart Hospital, Hallym University College of Medicine, Chuncheon, Republic of Korea; 4 Department of Neurology, Severance Hospital, Yonsei University College of Medicine, Seoul, Republic of Korea; 5 Department of Neurology, Dongtan Sacred Heart Hospital, Hallym University College of Medicine, 7 Keunjaebong-Gil, Hwaseong, 18450 Republic of Korea; 6 Department of Neurology, Inje University Ilsan Paik Hospital, Inje University College of Medicine, Goyang, Republic of Korea; 7 Department of Neurology, Uijeongbu Eulji Medical Center, Eulji University School of Medicine, Uijeongbu, Republic of Korea; 8 Department of Neurology, Jeonbuk National University Hospital, Jeonbuk National University School of Medicine, Jeonju, Republic of Korea; 9 Department of Neurology, School of Medicine, Kyungpook National University, Daegu, Republic of Korea; 10 Department of Neurology, Yongin Severance Hospital, Yonsei University College of Medicine, Yongin, Republic of Korea; 11 Department of Neurology, Kangdong Sacred Heart Hospital, Hallym University College of Medicine, Seoul, Republic of Korea; 12 Department of Neurology, Seoul National University Hospital, Seoul National University College of Medicine, 101, Daehak-Ro Jongno-Gu, Seoul, 03080 Republic of Korea

**Keywords:** Asian, Intracranial hypertension, Intracranial pressure, Papilledema, Pseudotumor cerebri

## Abstract

**Background:**

Currently, there is a relative lack of detailed reports regarding clinical presentation and outcome of idiopathic intracranial hypertension in Asians. This study aims to describe the clinical features and treatment outcomes of Korean patients with idiopathic intracranial hypertension.

**Methods:**

We prospectively recruited patients with idiopathic intracranial hypertension from one hospital and retrospectively analyzed the medical records of 11 hospitals in Korea. We collected data regarding preceding medical conditions or suspected medication exposure, headache phenotypes, other associated symptoms, detailed neuroimaging findings, treatments, and outcomes after 1–2 and 3–6 months of treatment.

**Results:**

Fifty-nine (83.1% women) patients were included. The mean body mass index was 29.11 (standard deviation, 5.87) kg/m^2^; only 27 patients (45.8%) had a body mass index of ≥ 30 kg/m^2^. Fifty-one (86.4%) patients experienced headaches, patterns of which included chronic migraine (15/51 [29.4%]), episodic migraine (8/51 [15.7%]), probable migraine (4/51 [7.8%]), chronic tension-type headache (3/51 [5.9%]), episodic tension-type headache (2/51 [3.9%]), probable tension-type headache (2/51 [3.9%]), and unclassified (17/51 [33.3%]). Medication overuse headache was diagnosed in 4/51 (7.8%) patients. After 3–6 months of treatment, the intracranial pressure normalized in 8/32 (25.0%), improved in 17/32 (53.1%), no changed in 7/32 (21.9%), and worsened in none. Over the same period, headaches remitted or significantly improved by more than 50% in 24/39 patients (61.5%), improved less than 50% in 9/39 (23.1%), and persisted or worsened in 6/39 (15.4%) patients.

**Conclusion:**

Our findings suggest that the features of Asian patients with idiopathic intracranial hypertension may be atypical (i.e., less likely obese, less female predominance). A wide spectrum of headache phenotypes was observed. Medical treatment resulted in overall favorable short-term outcomes; however, the headaches did not improve in a small proportion of patients.

## Background

Idiopathic intracranial hypertension (IIH) is a rare condition characterized by increased cerebrospinal fluid (CSF) pressure, in the absence of ventriculomegaly, mass lesions, underlying infection, or malignancy [1, 2], which was previously known as benign intracranial hypertension or pseudotumor cerebral syndrome. Despite its benign nomenclature, IIH can cause severe headaches, vision disturbance or loss, and severe functional impairment, necessitating invasive procedures such as CSF shunting or bariatric surgery [3–7].

The criteria for diagnosing increased CSF pressure have evolved over time. The International Classification of Headache Disorders (ICHD), 2nd Edition, published in 2004, defined cut-off values of IIH as 20.0 cmCSF in non-obese and 25.0 cmCSF in obese individuals. The International Classification of Headache Disorders, 3rd Edition (ICHD-3), published in 2018, revised these values to 25.0 cmCSF for the general population and 28.0 cmCSF for obese children [8–11]. Concurrently, obesity, defined as body mass index (BMI) of 30 or more, had prevalence rates of 5.2% and 7.3% in Korea and 42.4% and 42.0% in the United States in 2018 and 2022, respectively. Significant correlations between IIH and both obesity and socioeconomic disadvantage have been documented [12–15], suggesting that relying solely on CSF pressure for the diagnosis might be insufficient [16].

Ophthalmological and neuroradiological findings can be used to support the diagnosis of IIH. Papilledema with normal brain parenchyma and absence of hydrocephalus is a hallmark of IIH. A combination of several imaging findings has been recognized as capable of providing supportive evidence of IIH, including empty sella, flattening of the posterior aspect of the globe, distention of the perioptic subarachnoid space with or without a tortuous optic nerve, and transverse venous sinus stenosis [17, 18]. In cases lacking papilledema, IIH without papilledema (IIHWOP) is diagnosed based on unilateral or bilateral sixth cranial nerve palsy, and a minimum of three neuroradiological findings [3]. Moreover, a diagnosis of probable IIH is considered when papilledema is present, but CSF pressure is below these thresholds, as per the criteria updated by Friedman [1].

A discrepancy in IIH incidence between regions has been noted, with research showing a markedly lower prevalence in Asia than in Europe and North America [12]. The incidence of IIH in Japan has been estimated as 0.03 per 100,000 [19]. In contrast, the incidence of IIH increased threefold from 2.3 per 100,000 in 2003 to 7.8 per 100,000 in 2017 in England [5, 20, 21]. This variance could be attributed to genuine regional differences in obesity rates, potential underdiagnosis of IIH in Asian patients due to the nuanced clinical presentation of IIH, or diagnostic criteria that may require regional validation. Detailed information from a broader spectrum of patients with IIH is required to fill this knowledge gap.

In this context, the present multicenter study aims to delineate the clinical profile and treatment outcomes of IIH in Korea.

## Methods

### Study setting

We retrospectively identified patients with IIH from the medical records of 11 hospitals, and concurrently collected data from a prospective headache registry in one hospital from January 2019 to December 2021. The inclusion criteria were modified from the diagnostic criteria revised by Friedman to encompass a broader spectrum of IIH manifestations [1], including milder elevation of intracranial pressure (ICP). Our working criteria for diagnosis of IIH were as follows: presence of either 1) CSF opening pressure of 25.0 cmCSF or greater on lumbar puncture or 2) opening pressure of 20.0 ~ 24.9 cmCSF, with at least one of the following: papilledema, unilateral or bilateral 6th nerve palsy, and neuroimaging findings suggestive of IIH including empty sella, flattening of the posterior globe, enlarged perioptic subarachnoid space and/or tortuous optic nerve, and transverse sinus stenosis. Patients with structural lesions that could have caused intracranial hypertension were also excluded.

The Institutional Review Board (IRB) of each participating hospital approved this study, for which the IRB number of the principal investigator was 2022AN0015. In one hospital, following a comprehensive explanation of the study details, written informed consent was obtained from all participants for the prospective design. In the remaining hospitals, the need for informed consent was waived by the ethics committee as the study comprised only a retrospective review of patient charts.

### Clinical manifestation and laboratory findings

We collected data on preexisting medical conditions, including migraine, anemia, hypertension, polycystic ovary syndrome, sleep apnea, renal failure, chronic obstructive pulmonary disease, systemic lupus erythematosus, pregnancy, adrenal insufficiency, Cushing’s syndrome, hypo- or hyperthyroidism, hypoparathyroidism, Down syndrome, Turner syndrome, and craniosynostosis. Medication exposure within 1 year prior to the diagnosis was also collected, including systemic steroids, fluoroquinolones, tetracycline class antibiotics, levothyroxine, cyclosporine, tamoxifen, danazol, vitamin A derivatives, nalidixic acid, levonorgestrel implant, lithium, growth hormone, indomethacin, and cimetidine.

We identified patient characteristics and phenotypic ICHD-3 classification of headache (migraine, tension-type, or other phenotype; episodic, chronic, vs. probable), and assessed medication overuse within three months prior to the diagnosis. Associated symptoms, including pulsatile tinnitus, transient visual obscuration, blurred vision or visual impairment, dizziness, double vision, photophobia, cognitive impairment, 6th nerve palsy, and neck or back pain, were also recorded. Additionally, we collected findings of CSF examinations, brain magnetic resonance imaging (MRI), and structural and functional tests of the optic nerves (e.g., fundus photography, optical coherence tomography, transorbital sonography, and perimetry). Based on these clinical and laboratory observations, we determined whether patients met the ICHD-3 criteria 7.1.1. Headache attributed to IIH.

### Treatment

We reviewed the medications prescribed for IIH treatment, documented doses, and patient responses from the medical records. Procedures, including repetitive CSF drainage, lumbar drainage, optic nerve sheath penetration, shunting, and venous sinus stenting [22], and patient responses were also collected if performed. Treatment responses were categorized by the physicians as effective, partially effective, or ineffective based on their clinical judgment.

### Outcome

We extracted medical records at 1 month (between 1 and 2 months) and at the second follow-up (between 3 and 6 months). For these two time points, we collected data on the modality used for the ICP follow-up and results, headache outcome (remission, ≥ 50% reduction in headache days, < 50% improvement, and no change/worsening), days with headache, moderate or severe headache, and acute medication use for the recent one month, changes in visual acuity and field defect if impaired at baseline, and overall impression of treatment outcomes (normalized, improved, no change, or worsened).

### Statistical analysis

We summarized the descriptive data regarding demographics, clinical profiles, headache profiles, associated symptoms, treatment modalities and responses, and clinical outcomes in terms of ICP and headache. Data are presented as number (percentage), mean ± standard deviation (SD), or median (interquartile range, IQR), depending on the data distribution. Data were evaluated for normality using the Shapiro–Wilk test. Statistical significance was set at *P* < 0.05. difference. Statistical analyses were performed using the SPSS software (version 20.0; IBM Corp., Armonk, NY, USA).

## Result

### Patient profiles

**Fig. 1:**
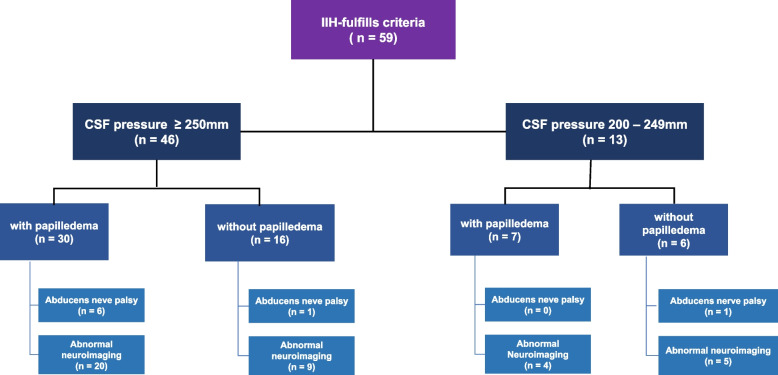
Flowchart of patient enrollment. A total of 59 patients with idiopathic intracranial hypertension fulfilled the inclusion criteria. Based on the lumbar puncture pressure, 46 patients (78.0%) were classified into the group with lumbar puncture pressure > 25.0 cmCSF. Among these, 62.5% had papilledema. Further, 13 patients (22.0%) were classified into the group with lumbar puncture pressure between 20.0 and 24.9 cmCSF, and 53.8% of this group had papilledema

A total of 59 patients diagnosed with IIH were included in this study, of whom 83.1% were women. Among these patients, 45 (78.0%) exhibited a lumbar puncture pressure > 25.0 cmCSF, while the remaining 13 (22.0%) had lumbar puncture pressure ranging from 20.0 and 24.9 cmCSF. The distribution of the patients according to the inclusion criteria is shown in Fig. 1.

The median age at diagnosis was 32.0 (IQR, 14 – 77) years. The mean BMI was 29.11 kg/m^2^ (SD, 5.87), and 27 patients (45.8%) had a BMI of 30 or more. Of the 59 patients with IIH, 26 (44.1%) had a history of migraine, 11 (18.6%) had hypertension, and 7 (11.6%) had anemia. In addition, among the female participants, eight (16.3%) reported a history of polycystic ovary syndrome. The most common medication exposure within the one year prior to presentation was systemic steroids (*n *= 9, 15.3%). The demographic data and clinical characteristics of the study population are summarized in Table 1.

**Table 1:** Demographic characteristics of idiopathic intracranial hypertension in Korea

Variables	All participants (*n* = 59)
Women**, n (%)**	49 (83.1%)
Age at diagnosis, years (median, IQR)	32.0 (14 – 77)
Age at registry enroll, years (median, IQR)	33.0 (17 – 84)
BMI, kg/m^2^ (mean ± SD)	29.11 ± 5.87
Current smoking	4 (6.8%)
Ex-smoker	2 (3.4%)
Non-smoker	53 (89.8%)
Alcohol	11 (18.6%)
Past medical history	
**Migraine**	26 (44.1%)
Hypertension	11 (18.6%)
Anemia	7 (11.9%)
Sleep apnea	3 (5.1%)
**Systemic lupus erythematous**	1 (1.7%)
Hyperthyroidism	1 (1.7%)
Hypothyroidism	3 (5.1%)
Polycystic ovary syndrome^**a**^	8 (16.3%)
Pregnancy^**a**^	3 (6.1%)
**Medication exposure within 1 year**	
Steroid	9 (15.3%)
Fluoroquinolones	1 (1.7%)
Levothyroxine	1 (1.7%)
Tamoxifen	1 (1.7%)

*IQR* interquartile range, *BMI* body mass index, *SD* standard deviation

^a^A proportion among female patients

### Characteristics of headache

Fifty-one patients (86.4%) with IIH had headaches; the clinical characteristics of these headache cases are summarized in Table 2. The median visual analog scale (VAS) score was 7.0 (IQR 5.8– 8.0), and 39 patients (76.5%) had moderate to severe headache. Forty-five patients (88.2%) had at least one headache attack in the month preceding their diagnosis. The median number of headache days within this period was 29, with patients experiencing a median of 11 days of moderate-to-severe headache and 5 days of acute headache medication. Four (7.8%) patients were diagnosed with medication-overuse headache.

**Table 2:** Clinical features of headache in 51 patients with idiopathic intracranial hypertension

Features of headache	*n* = 51
**Location**	
Unilateral	7 (13.7%)
Bilateral	44 (86.3%)
**VAS score**^**a**^	7.0 (5.8 – 8.0)
**Attack during recent 1 month**	
Presence of **h**eadache^b^	45 (88.2%)
**P**resence of moderate to severe **h**eadache^d^	39 (76.5%)
Days of headache^c^	29.0 (15.0 – 30.0)
Days of moderate to severe headache^d^	11 (5.0 – 27.5)
Days with acute headache medication^e^	5 (0.0 – 10.0)
**Medication-overuse headache within 3 months**^**f**^	4 (7.8%)
**Phenotypic ICHD-3 classification**	
Migraine**-like**	27 (52.9%)
Episodic	8 (15.7%)
Chronic	15 (29.4%)
Probable	4 (7.8%)
Tension-type headache**-like**	7 (13.7%)
Episodic	2 (3.9%)
Chronic	3 (5.9%)
Probable	2 (3.9%)
**Unclassified**	17 (33.3%)

Data were presented as frequencies (percentages) for categorical variables and medians (interquartile ranges) for continuous variables. The numbers of respondents differed for each item: 50 (a), 46 (b), 45 (c), 26 (e), and 31 (f)

*VAS* visual analog scale, *ICHD-3* International Classification of Headache Disorders, 3rd edition

Among 51 patients with headaches, 27 (52.9%) had a migraine-like headache feature, seven (13.7%) had a tension-type headache-like features, and 17 (33.3%) had unclassified headaches. Eighteen (35.3%) patients were categorized as having either chronic migraine or chronic tension-type headache. Overall, 45 out of 59 patients with IIH (76.3%) met the ICHD-3 criteria for headaches attributed to IIH.

### Non-headache clinical presentation

Of all patients diagnosed with IIH, 54 (91.5%) reported experiencing at least one clinical symptom associated with IIH, aside from headache. The most common non-headache symptoms were blurred vision or visual impairment (*n* = 30, 50.8%), followed by dizziness (*n* = 18, 30.5%), pulsatile tinnitus (*n* = 15, 25.4%), and transient visual obscuration (*n* = 13, 22.0%). Among 30 patients with blurred vision or visual impairment, six (20%) had transient visual obscuration and 24 experienced no transient visual obscuration events. The associated symptoms are summarized in Table 3.

**Table 3:** Non-headache presentation of idiopathic intracranial hypertension

Characteristics of symptoms	n (%)
Blurred vision or visual impairment	30 (50.8%)
Dizziness	18 (30.5%)
Pulsatile tinnitus	15 (25.4%)
**Double vision or diplopia**	14 (23.7%)
Transient visual obscuration	13 (22.0%)
Photophobia	11 (18.6%)
**Abducents nerve palsy**	8 (13.6%)
Neck pain	8 (13.6%)
Cognitive impairment	4 (6.8%)

Among the 51 patients with headaches, 12 had transient visual obscuration, 26 had blurred vision or visual impairment, 15 had dizziness, 11 had double vision, and 6 had cranial nerve VI palsy. However, no significant differences in these cranial nerve-related symptoms were found between patients with and without headaches, as determined by chi-square analysis (Table 4).

**Table 4:** Comparisons of associated symptoms between patients with and without headache

	**Headache ( +) (*n* = 51)**	**Headache (-) (*n* = 8)**	***p*-value**
Transient visual obscuration	12 (23.5%)	1 (12.5%)	0.671
Blurred vision	26 (51.0%)	4 (50.0%)	> 0.999
Dizziness	15 (29.4%)	3 (37.5%)	0.690
Double vision	11 (21.6%)	3 (37.5%)	0.379
CN VI palsy	6 (11.8%)	1 (12.5%)	0.295

### Laboratory, ophthalmologic, and neuroimaging findings

All patients underwent a lumbar puncture and fundus examination as part of the diagnostic process. The median lumbar puncture pressure was 27.2 cmCSF (IQR 25.5 – 34.0). The median white blood cell count was 1 (IQR 0 – 2) and the median protein level was 65.5 mg/dL (IQR 60.0 – 71.3). Fundus examination results were normal in 22 patients (37.3%), while Frisen papilledema grades 1–5 were as follows: grade 1, 15 patients (25.4%); grade 2, 4 patients (6.8%); grade 3, 7 patients (11.9%); grade 4, 8 patients (13.6%); and grade 5, 3 patients (5.1%).

In the MRI findings of the 22 patients with normal fundus examinations, the results varied; eight (36.4%) showed no abnormalities, five (22.7%) had findings consistent with empty sella, and two (9.1%) exhibited transverse sinus stenosis. Tortuous optic nerve and optic nerve cupping were each identified in one patient (4.5%). Three patients demonstrated two concurrent MRI abnormalities: empty sella with transverse sinus stenosis in two patients (9.1%) and a combination of tortuous optic nerve with transverse sinus stenosis in one patient (4.5%). Additionally, two patients (9.1%) exhibited a triad of abnormalities: empty sella, tortuous optic nerve, and transverse sinus stenosis.

Brain MRI scans with or without contrast enhancement were conducted in 58 patients (98%), revealing an empty sella in 16 patients (27.6%). Additionally, 22 patients (37.9%) underwent brain MRI with optic nerve thin-section imaging, identifying tortuous optic nerve in four patients (18.2%), optic nerve cupping in two patients (9.1%), and both findings in three patients (13.6%). MR venography was further performed in 33 patients (55.9%), with 19 patients (57.6%) showing transverse sinus stenosis.

### Treatment

In this study, 56 patients were treated with medication or other procedures. Of these, 50 (89.3%) were treated with acetazolamide at a median dose of 750 mg (range, 250 to 3000 mg). Additionally, 11 (19.6%) were administered topiramate, and 17 (30.3%) were prescribed migraine preventive medications. Thirteen patients (23.2%) had undergone temporary or repeated lumbar drainage as part of their treatment regimen.

### Outcomes

**Fig. 2:**
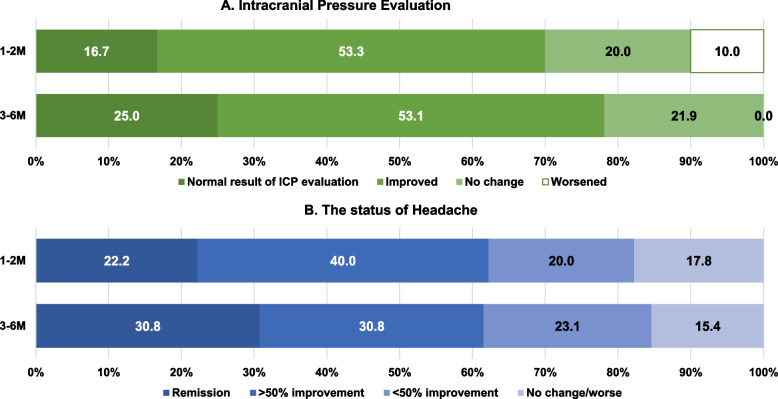
Outcomes of idiopathic intracranial hypertension. **A** Intracranial pressure evaluation at 1–2 months (*n* = 30) and 3–6 months (*n* = 32). **B** Headache status at 1–2 months (*n* = 45) and 3–6 months (*n* = 39) in patients with idiopathic intracranial hypertension who reported having a headache (*n* = 51)

Of the 59 patients with IIH, 55 (93.2%) underwent follow-up 1–2 months after their initial diagnosis. During this period, 30 patients (54.5%) underwent evaluation of ICP using methods such as lumbar puncture, optical coherence tomography, and fundus photography (Fig. 2A). The ICP was normalized in five patients (16.7%), was improved in 16 patients (53.3%), unchanged in six patients (20.0%), and worsened in three patients (10.0%).

Follow-up was performed between 3–6 months after diagnosis in 45 patients (76.3%). Among them, 32 (71.1%) underwent follow-up evaluation for ICP, with eight (25.0%) showing normalization, 17 (53.1%) demonstrating improvement, and ICP remaining unchanged in seven patients (21.9%). None of the patients exhibited worsened ICP during the follow-up period.

Of the 51 patients with IIH who reported experiencing headache, the headache status was assessed in 45 patients at the 1–2 months follow-up. Of the patients, 10 (22.2%) were in remission and 18 (40.0%) showed over 50% improvement, while eight (17.8%) had no interval change or worsened (Fig. 2B). At the 3–6 months follow-up, conducted among 39 patients, 24 (61.5%) of patients were in remission status or showed over 50% improvement, while six patients (15.4%) had no interval change or worsening in their headache status (Fig. 2B).

## Discussion

This multicenter investigation details the clinical, ophthalmological, and neuroimaging characteristics of IIH in a Korean cohort. Our main findings are as follows: 1) Our patients had a less prominent obesity and female predominance compared to European and North American cohorts; 2) patients showed diverse headache patterns from episodic tension-type headache, chronic migraine, and medication-overuse headache; and 3) medical treatment yielded overall favorable treatment outcome on ICP evaluation, but approximately 15% of patients did not show improvement in their headache during follow-up.

To our knowledge, this is the largest multicenter study thus far to describe the detailed clinical characteristics, especially the neuroimaging findings, of patients with IIH in Asia. To date, there have been four case series on IIH in Asia [19, 23–25]. In all previous series, the number of patients was small: 9 and 12 in two Chinese series, 2 in a Japanese series, and 10 in a Korean series. In our study, the mean BMI was 29 kg/m^2^, consistent with previous Asian studies but substantially lower than the 40 kg/m^2^ reported in the idiopathic intracranial hypertension treatment trial, which predominantly involved Caucasian or African American populations [26]. In addition, our study reported a relatively low percentage of women (83.1%) in contrast to the higher rates of over 90% observed in most European and North American IIH studies [27–32]. Taken together, so-called “atypical IIH” [33], i.e. IIH in non-obese and male patients, was not uncommon in our cohort, which represent the largest Asian cohort reported so far. Our findings emphasize the need for clinicians to adopt a broader diagnostic perspective, especially in Asian populations where IIH may not conform to the typical presentations observed in Western contexts.

The headache patterns were diverse in our study. Headache was most common symptom, reported by 86% of patients; however, the phenotypes and chronicities of these headaches varied widely among individuals. Contrary to the prevalent assumption that secondary headache disorders usually present with daily persistent headaches, episodic headaches with migraine or tension-type phenotypes also occurred, similar to the findings of the idiopathic intracranial hypertension treatment trial (IIHTT) [34]. This suggests that the characteristics and severity of headaches may not be definitive in the diagnosis of IIH. Notably, blurred vision or visual impairment was less common in our study than in IIHTT, probably due to the difference in the inclusion criteria (mild papilledema was mandatory in IIHTT) [29, 30], such as the requirement of mild papilledema in IIHTT. The clinical course of headaches associated with IIH after treatment also varied in this study.

Here, we describe detailed laboratory and neuroimaging findings in patients with IIH. Papilledema was present in only two-thirds of patients. In the remaining patients, brain MRI was crucial for the diagnosis of IIH, as was indicated in the latest criteria of Friedman et al. [1]. Brain MRI with optic nerve thin-section images and MR venography should be performed to screen for IIH, especially in Asian patients, as atypical clinical profiles may be more common in Asian patients.

In terms of treatment modality and outcome, the majority of patients received medical treatment with acetazolamide, with approximately 86% reporting either effective or partial effective response. No surgical treatment was required, and no patients reported any visual loss. At 6-month follow-up, 25% had achieved ICP normalization and approximately 50% showed improved outcomes. Taken together, IIH has a benign course, and acetazolamide may be an effective treatment option in our cohort. However, approximately 15% of patients reported no change or worsening of headache status at follow-up between 3–6 months. The post-IIH headache and the role of calcitonin gene-related peptide monoclonal antibody therapy should be considered in selected cases [35, 36]. Nonpharmacological approaches to controlling IIH and headaches, such as diet restriction and weight loss, have also been reported to be effective in obese children or adults [37, 38].

The strengths of this study include its multicenter-based approach and the analysis of the largest number of patients in Asia to date. Additionally, the low proportion of patients with vision loss and favorable prognosis are likely characteristics of intracranial hypertension in Asia. Finally, more than half of the patients had a BMI of less than 30, indicating that the importance of exploring risk factors other than obesity may need to be considered in Asian patients with IIH. However, this study has some limitations. Firstly, analysis in some centers was retrospective, and this study faced challenges with unstructured follow-up methods and timing. However, the follow-up completion rate was comparable to that reported in other prospective studies. Therefore, certain clinical features were not observed in this study. Nonetheless, considering the low prevalence of IIH in Asia, this study is valuable in presenting diverse headache patterns and non-headache presentations, treatment, and relatively benign outcomes in a broader spectrum of patients with IIH using multicenter data.

## Data Availability

The research data is not publicly available but is held by the authors and can be provided upon request.

## Abbreviations

IIH: Idiopathic intracranial hypertension
CSF: Cerebrospinal fluid
BMI: Body mass index
IIHWOP: IIH without papilledema
ICP: Intracranial pressure
MRI: Magnetic resonance imaging
SD: Standard deviation
IQR: Interquartile range
VAS: Visual analog scale
ICHD: The International Classification of Headache Disorders
IIHTT: Idiopathic intracranial hypertension treatment trial

## References

[1] Friedman DI, Liu GT, Digre KB (2013). Revised diagnostic criteria for the pseudotumor cerebri syndrome in adults and children. Neurology.

[2] McCluskey G, Doherty-Allan R, McCarron P, Loftus AM, McCarron LV, Mulholland D, McVerry F, McCarron MO (2018). Meta-analysis and systematic review of population-based epidemiological studies in idiopathic intracranial hypertension. Eur J Neurol.

[3] Mollan SP, Davies B, Silver NC, Shaw S, Mallucci CL, Wakerley BR, Krishnan A, Chavda SV, Ramalingam S, Edwards J (2018). Idiopathic intracranial hypertension: consensus guidelines on management. J Neurol Neurosurg Psychiatry.

[4] Best J, Silvestri G, Burton B, Foot B, Acheson J (2013). The incidence of blindness due to idiopathic intracranial hypertension in the UK. Open Ophthalmol J.

[5] Miah L, Strafford H, Fonferko-Shadrach B, Hollinghurst J, Sawhney IMS, Hadjikoutis S, Rees MI, Powell R, Lacey A, Pickrell WO (2021). Incidence, prevalence, and health care outcomes in idiopathic intracranial hypertension: a population study. Neurology.

[6] Mikkilineni S, Trobe JD, Cornblath WT, De Lott L (2019). Visual field mean deviation at diagnosis of idiopathic intracranial hypertension predicts visual outcome. J Neuroophthalmol.

[7] Yoo YJ, Yang HK, Choi JY, Kim JS, Hwang JM (2020). Neuro-ophthalmologic findings in visual snow syndrome. J Clin Neurol.

[8] Arnold M (2018). Headache classification committee of the international headache society (IHS) the international classification of headache disorders. Cephalalgia.

[9] Headache Classification Subcommittee of the International Headache Society (2004). The international classification of headache disorders. Cephalalgia.

[10] Yri HM, Jensen RH (2015). Idiopathic intracranial hypertension: clinical nosography and field-testing of the ICHD diagnostic criteria. A case-control study. Cephalalgia.

[11] Korsbæk JJ, Jensen RH, Høgedal L, Molander LD, Hagen SM, Beier D (2023). Diagnosis of idiopathic intracranial hypertension: a proposal for evidence-based diagnostic criteria. Cephalalgia.

[12] Hales CM, Fryar CD, Ogden CL. Prevalence of obesity and severe obesity among adults: United States, 2017–2018. https://www.cdc.gov/nchs/data/databriefs/db360-h.pdf. Accessed 30 Mar 2024 32487284

[13] Nam GE, Kim Y-H, Han K, Jung J-H, Rhee E-J, Lee W-Y (2021). Obesity fact sheet in Korea, 2020: prevalence of obesity by obesity class from 2009 to 2018. J Obesity Metab Syndr.

[14] Rilley L. Percentage of adult aged 18+ years with a body mass index (BMI) of 30kg/m2 or higher. [cited 2024.3.30]

[15] Collaboration NCDRF (2024). Worldwide trends in underweight and obesity from 1990 to 2022: a pooled analysis of 3663 population-representative studies with 222 million children, adolescents, and adults. Lancet.

[16] Kaipainen AL, Martoma E, Puustinen T, Tervonen J, Jyrkkänen H-K, Paterno JJ, Kotkansalo A, Rantala S, Vanhanen U, Leinonen V (2021). Cerebrospinal fluid dynamics in idiopathic intracranial hypertension: a literature review and validation of contemporary findings. Acta Neurochir.

[17] Morris P, Black D, Port J, Campeau N (2017). Transverse sinus stenosis is the most sensitive MR imaging correlate of idiopathic intracranial hypertension. Am J Neuroradiol.

[18] Maralani P, Hassanlou M, Torres C, Chakraborty S, Kingstone M, Patel V, Zackon D, Bussière M (2012). Accuracy of brain imaging in the diagnosis of idiopathic intracranial hypertension. Clin Radiol.

[19] Yabe I, Moriwaka F, Notoya A, Ohtaki M, Tashiro K (2000). Incidence of idiopathic intracranial hypertension in Hokkaido, the northern-most island in Japan. J Neurol.

[20] Kilgore KP, Lee MS, Leavitt JA, Mokri B, Hodge DO, Frank RD, Chen JJ (2017). Re-evaluating the incidence of idiopathic intracranial hypertension in an era of increasing obesity. Ophthalmology.

[21] Sundholm A, Burkill S, Waldenlind E, Bahmanyar S, Nilsson Remahl AIM (2021). A national Swedish case-control study investigating incidence and factors associated with idiopathic intracranial hypertension. Cephalalgia.

[22] Jung H-J, Jung M, Park KY, Chang JY (2022). Effect of transverse sinus stenting on diffuse leukoencephalopathy with idiopathic intracranial hypertension. J Korean Neurol Assoc.

[23] Liu IH, Wang AG, Yen MY (2011). Idiopathic intracranial hypertension: clinical features in Chinese patients. Jpn J Ophthalmol.

[24] Kim TW, Choung HK, Khwarg SI, Hwang JM, Yang HJ (2008). Obesity may not be a risk factor for idiopathic intracranial hypertension in Asians. Eur J Neurol.

[25] Chen Q, Feng C, Zhao G, Chen W, Wang M, Sun X, Sha Y, Li Z, Tian G (2020). Pseudotumour cerebri syndrome in China: a cohort study. Sci Rep.

[26] Committee NIIHSGW, Wall M, McDermott MP, Kieburtz KD, Corbett JJ, Feldon SE, Friedman DI, Katz DM, Keltner JL, Schron EB, Kupersmith MJ (2014). Effect of acetazolamide on visual function in patients with idiopathic intracranial hypertension and mild visual loss: the idiopathic intracranial hypertension treatment trial. JAMA.

[27] Blanch R, Vasseneix C, Liczkowski A, Yiangou A, Aojula A, Micieli J, Mollan S, Newman N, Biousse V, Bruce B (2019). Differing presenting features of idiopathic intracranial hypertension in the UK and US. Eye.

[28] Volpe NJ (2014). Idiopathic intracranial hypertension: important questions answered with more to come. JAMA Neurol.

[29] Wall M, George D (1991). Idiopathic intracranial hypertension: a prospective study of 50 patients. Brain.

[30] Corbett JJ, Savino PJ, Thompson HS, Kansu T, Schatz NJ, Orr LS, Hopson D (1982). Visual loss in pseudotumor cerebri: follow-up of 57 patients from five to 41 years and a profile of 14 patients with permanent severe visual loss. Arch Neurol.

[31] Corbett JJ (2008). Familial idiopathic intracranial hypertension. J Neuroophthalmol.

[32] Kharode C, McAbee G, Sherman J, Kaufman M (1992). Familial intracranial hypertension: report of a case and review of the literature. J Child Neurol.

[33] Bruce B, Kedar S, Van Stavern G, Corbett J, Newman N, Biousse V (2010). Atypical idiopathic intracranial hypertension: normal BMI and older patients. Neurology.

[34] Friedman DI, Quiros PA, Subramanian PS, Mejico LJ, Gao S, McDermott M, Wall M, Group NIS (2017). Headache in idiopathic intracranial hypertension: findings from the idiopathic intracranial hypertension treatment trial. Headache.

[35] Yiangou A, Mitchell JL, Fisher C, Edwards J, Vijay V, Alimajstorovic Z, Grech O, Lavery GG, Mollan SP, Sinclair AJ (2021). Erenumab for headaches in idiopathic intracranial hypertension: a prospective open-label evaluation. Headache.

[36] Mollan SP, Grech O, Sinclair AJ (2021). Headache attributed to idiopathic intracranial hypertension and persistent post-idiopathic intracranial hypertension headache: a narrative review. Headache.

[37] Na JH (2024). Application and effectiveness of dietary therapies for pediatric migraine. Headache Pain Res.

[38] Kupersmith MJ, Gamell L, Turbin R, Peck V, Spiegel P, Wall M (1998). Effects of weight loss on the course of idiopathic intracranial hypertension in women. Neurology.

